# Haemoglobin‐mediated response to hyper‐thermal stress in the keystone species *Daphnia magna*

**DOI:** 10.1111/eva.12561

**Published:** 2017-11-02

**Authors:** Maria Cuenca Cambronero, Bettina Zeis, Luisa Orsini

**Affiliations:** ^1^ Environmental Genomics Group School of Biosciences the University of Birmingham Birmingham UK; ^2^ Institute of Zoophysiology University of Muenster Muenster Germany

**Keywords:** conservation genetics, eutrophication, evolution, global warming, oxygen metabolism, plasticity, resurrection, thermal tolerance

## Abstract

Anthropogenic global warming has become a major geological and environmental force driving drastic changes in natural ecosystems. Due to the high thermal conductivity of water and the effects of temperature on metabolic processes, freshwater ecosystems are among the most impacted by these changes. The ability to tolerate changes in temperature may determine species long‐term survival and fitness. Therefore, it is critical to identify coping mechanisms to thermal and hyper‐thermal stress in aquatic organisms. A central regulatory element compensating for changes in oxygen supply and ambient temperature is the respiratory protein haemoglobin (Hb). Here, we quantify Hb plastic and evolutionary response in *Daphnia magna* subpopulations resurrected from the sedimentary archive of a lake with known history of increase in average temperature and recurrence of heat waves. By measuring constitutive changes in crude Hb protein content among subpopulations, we assessed evolution of the Hb gene family in response to temperature increase. To quantify the contribution of plasticity in the response of this gene family to hyper‐thermal stress, we quantified changes in Hb content in all subpopulations under hyper‐thermal stress as compared to nonstressful temperature. Further, we tested competitive abilities of genotypes as a function of their Hb content, constitutive and induced. We found that Hb‐rich genotypes have superior competitive abilities as compared to Hb‐poor genotypes under hyper‐thermal stress after a period of acclimation. These findings suggest that whereas long‐term adjustment to higher occurrence of heat waves may require a combination of plasticity and genetic adaptation, plasticity is most likely the coping mechanism to hyper‐thermal stress in the short term. Our study suggests that with higher occurrence of heat waves, Hb‐rich genotypes may be favoured with potential long‐term impact on population genetic diversity.

## 1 INTRODUCTION

Although variation in temperature has occurred throughout Earth's history, anthropogenic climate change has become a major geological and environmental force (Corlett, 2015; Parmesan, 2007; Parmesan & Yohe, 2003) driving drastic changes in natural ecosystems (Hoffmann & Sgro, 2011; Hoffmann et al., 2015; McGill, Dornelas, Gotelli, & Magurran, 2015). In particular, the speed and severity of change in temperature combined with the occurrence of extreme events like heat weaves (Moss, 2012) is a major threat to biodiversity (Urban, 2015) and community composition (Corlett, 2015).

Freshwater ecosystems are among the most impacted by global warming, both via direct changes in environmental temperatures and indirectly by changes in biotic and abiotic conditions, such as acidity, eutrophication, metal pollution, pesticide leaching from land use and UV radiation (Altshuler et al., 2011). In particular, changes in water temperature alter oxygen solubility with cascading effects on respiration and metabolic processes of ectotherms (Hochachka & Somero, 2002). Osmotic and metabolic stress, in turn, make water organisms more susceptible to other environmental challenges, such as chemical pollution (Altshuler et al., 2011; Ha & Choi, 2009) and pathogens (Garbutt, Scholefield, Vale, & Little, 2014; Schade, Shama, & Wegner, 2014).

The ectotherm *Daphnia* is present in the majority of lotic habitats including arctic and temperate lakes, lakes at high elevations and ephemeral ponds, where it plays a central role in the food chain (Miner, De Meester, Pfrender, Lampert, & Hairston, 2012). This ectotherm is exposed to severe spatial and temporal environmental changes, including temperature and oxygen. Previous studies on *Daphnia magna* provide strong evidence of evolution of temperature tolerance across few decades (Geerts et al., 2015). This evolution is mediated by both plastic and evolutionary changes in gene expression at a number of candidate genes, including some heat shock proteins (Jansen et al., 2017; Klumpen et al., 2017). However, the candidate genes identified are only indirectly linked to temperature (e.g., many identified genes are central metabolic or immune response genes). Other control systems to compensate for temperature variation in species of the genus *Daphnia* include physiological (Zeis et al., 2009), behavioural (Pirow, Bäumer, & Paul, 2001) and biochemical (Zeis, Becker, Gerke, Koch, & Paul, 2013; Zeis et al., 2003) mechanisms. A central regulatory element compensating for changes in oxygen supply and ambient temperature in *Daphnia* is the respiratory protein haemoglobin (Hb) (Paul, Zeis, Lamkemeyer, Seidl, & Pirow, 2004; Paul et al., 2004; Paul, Zeis, Maurer, Pinkhaus, Bongartz, & Paul, 2004; Gerke, Börding, Zeis, & Paul, 2011). Hb is an extracellular, multi‐subunit respiratory protein encoded by 11 genes, each consisting of two haeme‐containing globin domains (Gerke et al., 2011). Hb expression in *Daphnia* is regulated by the inducible factor (HIF‐1) (Tokishita et al., 1997) and hypoxia‐responsive elements located upstream of several Hb subunit genes (Gorr, Cahn, Yamagata, & Bunn, 2004). Changes in Hb concentration as well as Hb subunit composition modulate Hb oxygen affinity in *Daphnia*, guaranteeing oxygen supply to tissues (Gerke et al., 2011) and improving survival and activity under hypoxia (Kobayashi & Gonoi, 1985). The Hb protein has been shown to have significant clonal differences associated with oxygen tolerance (Weider, 1985) and temperature acclimation (Lamkemeyer, Zeis, & Paul, 2003) in *D*. *magna*. Moreover, oxygen consumption in *D. magna* has been shown to increase exponentially with ambient temperature (Paul, Zeis, et al., 2004; Paul, Lamkemeyer, et al., 2004). As tissue hypoxia is evoked by warm temperatures (Kobayashi, Fujiki, & Suzuki, 1988; Lamkemeyer et al., 2003), *Daphnia* organs increase oxygen demand to cope with thermal stress (Zeis et al., 2013). The evolution of Hb has been previously linked to physiological specialization of vertebrate species living in extreme environments [Wholly mammoth (Campbell et al., 2010)], providing evidence of the role of Hb in thermal adaptation across the animal kingdom.

Here, we study the role of Hb in the response to thermal and hyper‐thermal stress in the waterflea *D. magna*, a large‐bodied ectotherm common in European freshwater ecosystems (Miner et al., 2012). *Daphnia*'s parthenogenetic life cycle enables rearing populations of genetically identical individuals (clones) from a single genotype, providing the advantages of isogenic model organisms while retaining the natural genetic variation (Miner et al., 2012). As part of its life cycle, *Daphnia* produces dormant embryos, which remain viable in layered lake sediments for decades or even centuries (Frisch et al., 2014). These dormant embryos can be “resurrected” by the practise of resurrection ecology (Kerfoot & Weider, 2004) and maintained via clonal reproduction in the laboratory. Resurrection of historical populations, combined with the short generation time and clonal reproduction, provides the unique advantage of performing common garden experiments in which historical and modern populations’ performance in response to environmental change can be revealed. Collectively, the properties of *Daphnia* enable the contribution of plastic and genetic adaptive response to environmental stress to be disentangled by measuring evolutionary responses across multiple generations and fitness responses of the same genotype to multiple stressors.

To assess the evolutionary role of Hb in thermal tolerance, we resurrected dormant populations (1960–2005) of *D. magna* from a well‐characterized lake in Denmark (Orsini et al., 2016). Lake Ring has experienced an increase in average temperature and heat waves over time, especially in the last decades. Moreover, changes in water chemistry occurred because of a severe event of eutrophication (1960–1970) (Sayer, Davidson, & Jones, 2010) leading to reduced oxygen availability, from which the lake partially recovered in modern times. We measured constitutive (evolutionary) differences in Hb protein content among the subpopulations resurrected across these major environmental transitions, and linked these evolutionary differences to competitive abilities of genotypes under hyper‐thermal stress. We also quantified plastic response in Hb by exposing the genotypes from the three subpopulations to hyper‐thermal stress and quantifying changes in Hb protein content as compared to a nonstressful temperature regime. Our approach enabled us to assess the mechanisms of Hb evolution in response to higher occurrence of heat waves and increase in average temperature.

Specifically, we answered the following questions: (i) Has constitutive Hb protein content evolved over time in response to higher occurrence of heat waves and increase in average temperature? (ii) What mechanisms—plasticity, evolution or a combination thereof—underlie Hb response to hyper‐thermal stress? (iii) Are differences in constitutive Hb protein content among genotypes associated with superior competitive abilities in presence of extreme temperature?

By measuring constitutive differences in Hb protein content among subpopulations, we assessed whether evolution in this candidate gene family occurred in coincidence with an increase in average temperature and a higher occurrence of heat waves. By measuring differential Hb protein content between a nonstressful temperature regime (20°C) and hyper‐thermal stress (30°C), we quantified plasticity in this candidate gene family in response to hyper‐thermal stress. We then established a link between Hb levels and competitive abilities of genotypes. In common garden experiments, we assessed whether Hb‐rich genotypes had superior competitive abilities under hyper‐thermal stress in presence (microcosms) and absence (mesocosms) of prior acclimation to this stress.

## 2 MATERIALS AND METHODS

### 2.1 Study system

We study a population of *D. magna* resurrected from Lake Ring, a shallow mixed lake (maximum depth is 5 m) in Jutland, Denmark (55°57′51.83′′N, 9°35′46.87′′E) (Sayer et al., 2010). Lake Ring's history of anthropogenic impact is known from historical records (Berg, Jeppesen, Sondergaard, & Mortensen, 1994) and a previous palaeolimnological analysis conducted on a sedimentary archive sampled from the lake (Cambronero Cuenca & Orsini, 2017; Orsini et al., 2016). According to the weather station of Samsø, located 80 km from Lake Ring, the summer air temperature has experienced a steadily, even if modest (~1°C) increase in the last decades. The occurrence of heat waves in Europe has been documented by the IPCC (IPCC 2007). Because air and water surface temperature have a positive correlation for shallow streams and lakes (Preudhomme & Stefan, 1992), especially for the summer months (e.g., Livingstone & Lotter, 1998), we used the data from the weather station as a proxy for the monthly water temperature in Lake Ring. According to historical records, the lake experienced changes in water chemistry due to an event of severe eutrophication triggered by sewage inflow from a nearby town in 1960 (Michels, 2008). The sewage inflow was diverted from the lake in the late 1970s, but high fish stocking (Berg et al., 1994) and the use of pesticides due to agricultural intensification (Michels, 2008) maintained high levels of primary production until 1985. Finally, the lake partially recovered and returned to clear‐water conditions in modern times (>1999). Hence, based on historical and palaeolimnological records, the history of the lake consists of a period of severe eutrophication (1960–1970, eutrophication phase, EP), followed by pesticide leaching that maintained high trophic levels (1975–1985, pesticide phase) and a return to clear‐water conditions (clear‐water phase, CWP, >1999). From each lake phase, subpopulations of *D. magna* were resurrected from resting eggs, early stage embryos that arrest their development and are protected from the environment by a chitin case called ephippium (Ebert, 2005). The ephippia are easily spotted by eye in the sieved sediment, from where they are isolated and hatched by exposure to light and temperature stimuli (Cambronero Cuenca & Orsini, 2017). After hatching, the genotypes were kept in monoclonal cultures for several generations (up to a year) under standard laboratory conditions (10°C, long‐day photoperiod 14:10 L:D). Among the hatched embryos, which are all genetically distinct, we selected 10 random genotypes from each lake phase for a total of 30 genotypes. The genetic diversity for Lake Ring subpopulations is comparable to the one of other natural *D. magna* populations (Orsini et al., 2016). The sample size per subpopulation was chosen based on a previous study in which the threshold sample size and marker set required to assess genetic diversity in *D. magna* populations was assessed using a rarefaction analysis on the populations from three biological archives, including Lake Ring's, and on a set of 19 populations with relatively large sample size (Orsini et al., 2016). According to this study, 10–15 genotypes are a satisfactory representation of the genetic diversity of *D*. *magna* populations and subpopulations.

### 2.2 Haemoglobin evolutionary and plastic response

We measured constitutive Hb protein content in the 30 genotypes resurrected from the three subpopulations of *D. magna* using three technical replicates per genotypes. We measure Hb from crude extracts of animals reared in normoxic (saturated oxygen level) conditions at two experimental temperatures, 20 and 30°C. 20°C represents a nonstressful temperature regime whereas 30°C represents a hyper‐thermic stress that the animals may face under global warming with higher incidence of heat waves. To reduce interference from maternal effect, prior to Hb extraction, the animals were acclimated at the two temperatures in M4 medium (Elendt & Bias, 1990) for at least three generations in long light:dark photoperiod (16:8 hr) and fed ad libitum (2.5 mg C/L) *Desmodesmus subspicatus* (SAG 53.80, Göttingen, Germany). These culturing conditions were used throughout the experiments, including the competition experiments described below. Hb crude extracts were obtained from pools of ten individuals per genotype following established protocols (Schwerin, Zeis, Horn, Horn, & Paul, 2010). In brief, after removing adhering water, the pools of animals were weighed on a precision scale (BP 211D; Sartorius) and homogenized in 100 μl of M4 medium and mini complete protease inhibitor cocktail (Roche) using Teflon^®^ pestles (Kimble Kontes, Sigma Aldrich, Darmstadt, Germany). The homogenate was subjected to photometric measurements for protein determination using an Ultrospec 3000 (Pharmacia Biotech). The absorption spectrum was calculated for oxygenated and deoxygenated Hb between 250 and 800 nm for each sample. Deoxygenated samples were obtained by adding sodium dithionite crystals. This approach capitalizes on the property of Hbs to shift their absorbance maximum in presence and absence of oxygen bound as opposed to other compounds absorbing at these wavelengths which do not alter their absorbance. The spectrum area comprised between 422 and 452 nm was quantified for each sample using a standard purified Hb solution of known concentration as a reference following Schwerin (Schwerin et al., 2010).

Using a two‐way ANOVA, we measured whether changes in Hb protein content were explained by plasticity (response to treatment, temperature), evolution (differences among populations) or a combination thereof (treatment × population), using the lm function in R v.3.3.3 (R core team 2017).

### 2.3 Linking haemoglobin content to competitive performance under hyper‐thermal stress—mesocosms

To assess whether higher constitutive protein content provided superior competitive abilities under hyper‐thermal stress as compared to a nonstressful temperature regime, we performed a mesocosm competition experiment using 29 genotypes (one genotype went extinct in the pre‐exposure phase) from the three lake phases described above. The 29 genotypes were cultured in common garden conditions [25°C, light:dark photoperiod (16:8 hr) and fed ad libitum (2.5 mg C/L) *D. subspicatus*] in aerated M4 medium for at least three generations to reduce confounding factors due to maternal effect. Five juveniles of 24–48 hr from the second clutch of the third generation were randomly assigned to 12L mesocosms for a total of 145 animals per mesocosm [29 genotype × 5 juveniles × 2 mesocosms (20°C and 30°C) × 3 replicates]. The experimental mesocosms were maintained at the experimental temperatures for 4 weeks (≥3 clonal generations). To simulate a population dynamics that *Daphnia* may encounter in the natural environment, we culled 10% of the volume of each mesocosm at regular intervals on day 10, 17 and 24, after thorough mixing, removing medium and a random number of individuals collected in the culled medium. The culled volume (1.2L) was replenished with fresh medium in each mesocosm. At the end of the fourth week, 32 animals from each mesocosm (*N* = 192) were sampled to assess shifts in genotypic composition and frequency after selection as compared to the initial inoculum. To assess genotype frequency changes, we used a panel of 13 microsatellites arranged in two multiplexes (M01 and M05, Appendix S1). These loci are part of a panel of 84 microsatellites previously developed for *D. magna* (Jansen, Geldof, De Meester, & Orsini, 2011; Orsini, Spanier, & De Meester, 2012). Genomic DNA was extracted from single individuals using AGENCOURT^®^ DNAdvance (Beckman Coulter) kit with minor modifications. The samples were amplified using established protocols (Jansen et al., 2011; Orsini et al., 2012) and genotyped on an ABI3032. Fragment analysis was conducted with Genemapper (Thermo Fisher Scientific) using LIZ500 (Thermo Fisher Scientific) as size standard.

A chi‐square test was used to quantify differences in population frequency between the inoculum (29 genotypes × 5 juveniles) and the end of the experiment—4 weeks of treatment in each temperature. Population frequency differences between the two temperatures were also assessed with a chi‐square test.

### 2.4 Competitive abilities of the Hb‐rich genotypes after acclimation to hyper‐thermal stress—microcosms

To test whether prior acclimation to hyper‐thermal stress may enhance competitive abilities of the Hb‐rich genotypes, we performed microcosm experiments in which three randomly paired genotypes were competed, one having high constitutive Hb protein content and one having low constitutive Hb protein content (Appendix S2). These three random pairs were competed for 4 weeks after an initial acclimation of three generations to the experimental temperatures (20 and 30°C). Five juveniles of 24–48 hr from the second clutch of the third generation from each experimental temperature were used to inoculate 3 × 2.5 L microcosms of aerated M4 medium. After 4 weeks, 40 individuals were sampled from each microcosm and the genotypic composition determined using the diagnostic allozyme GOT (GOT; EC 2.6.1.1). Differences between the initial inoculum and the genotypes frequency after 4 weeks at both experimental temperatures were quantified using a chi‐square test.

### 2.5 Time to immobilization under hyper‐thermal stress

We measured temperature tolerance as knockout time (time to immobilization, *T*_imm_) on the three pairs of genotypes used in the microcosm experiment (Appendix S2) following Yampolsky, Schaer, & Ebert (2014). After three generations of acclimation to the experimental temperatures (20 and 30°C), three replicates of each genotype, each containing five adult females, were transferred into 1‐ml cuvette and heated at 37°C in a thermostatic photometer (Ultrospec 3000; Pharmacia Biotech). The *Daphnia*'s swimming activity was monitored continuously at 580 nm (Zeis et al., 2004). *T*_imm_ was recorded as the time elapsing between the exposures at 37°C until the time all five animals lost locomotion. To test the impact of temperature and Hb content on time to immobilization (*T*_imm_) at the two experimental temperatures and between Hb‐rich and Hb‐poor genotypes, we used a two‐way ANOVA performed using the lm function in R v.3.3.3 (R core team 2017).

## 3 RESULTS

### 3.1 Haemoglobin evolutionary and plastic response

**Figure 1:**
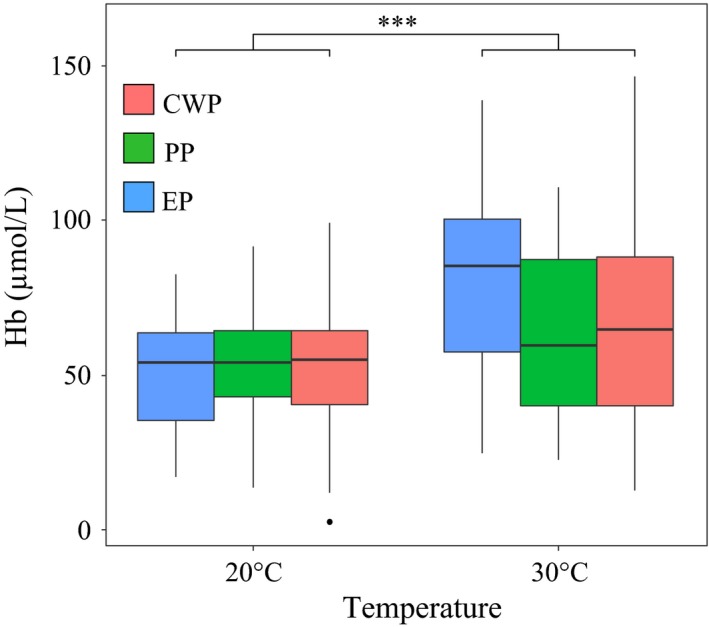
Constitutive haemoglobin differences among populations. Population‐averaged median and quartiles (25th and 75th) of haemoglobin (Hb) expression under hyper‐thermal stress (30°C) and a nonstressful temperature regime (20°C) in the subpopulations of *Daphnia magna* resurrected from Lake Ring, Denmark. EP—eutrophic subpopulation; PP—pesticide subpopulation; CWP—clear‐water subpopulation. The subpopulations are ordered on the *x*‐axis from historical to modern. The contribution of evolution (differences among populations), plasticity (response to treatment) and their interaction term was tested via ANOVA analysis (Appendix S3)

**Figure 2:**
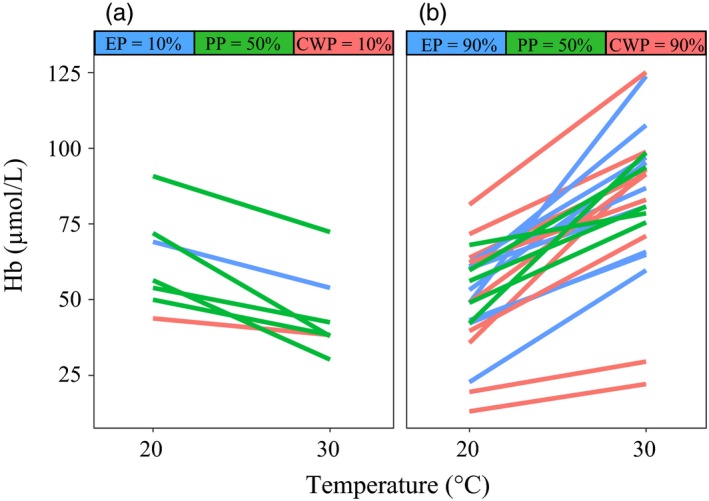
Haemoglobin expression under hyper‐thermal stress. Reaction norms of haemoglobin (Hb) expression per genotype under hyper‐thermal stress (30°C) as compared to a nonstressful temperature regime (20°C). (a) Genotypes downregulating and (b) upregulating haemoglobin under hyper‐thermal stress as well as the percentage of genotypes changing expression in each resurrected subpopulation are shown. The reaction norms are colour‐coded as in Figure 1

Nonsignificant evolutionary (constitutive) differences in Hb protein content were observed among the subpopulations of *D. magna* (Figure 1, Appendix S3). Conversely, significantly higher synthesis of Hb was observed under hyper‐thermal stress as compared to a nonstressful temperature (Figure 1). Specifically, 23% of the genotypes showed downregulation of Hb under hyper‐thermal stress (Figure 2a), whereas 76% showed upregulation (Figure 2b). The upregulation in Hb ranged between 0.04 and 1.6 log2 fold, whereas the downregulation ranged between 0.02 and 0.9 log2 fold (Appendix S4). The number of genotypes upregulating Hb was higher than the number of genotypes downregulating Hb in the eutrophic (EP) and clear‐water (CWP) subpopulations whereas an equal proportion of up and downregulating genotypes was present in the pesticide subpopulation (PP) (Figure 2). Significant population per temperature interaction was observed for constitutive difference in Hb (Appendix S3), likely driven by subpopulation specific response to hyper‐thermal stress (Figure 1).

### 3.2 Linking haemoglobin content to competitive performance under hyper‐thermal stress—mesocosms

**Figure 3:**
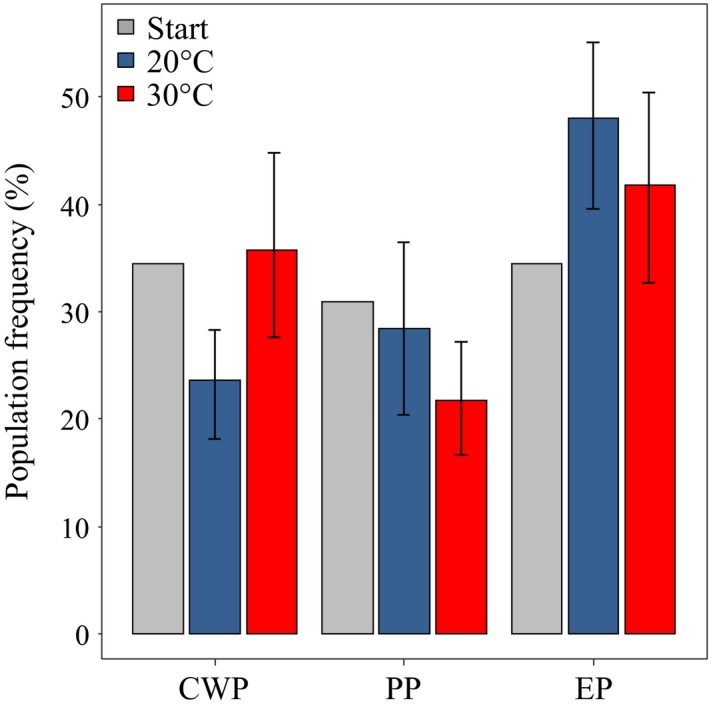
Population frequency after selection in mesocosm experiments. Changes in subpopulation frequency are shown after 4 weeks of selection at 20°C (blue bars) and 30°C (red bars) as compared to an initial inoculum (start) of equal frequency of the three subpopulations. Variance among replicate mesocosms is shown for the temperature regimes. The staring inoculum had no variance as all mesocosms are inoculated with equal number of genotypes. Population codes are as in Figure 1

**Figure 4:**
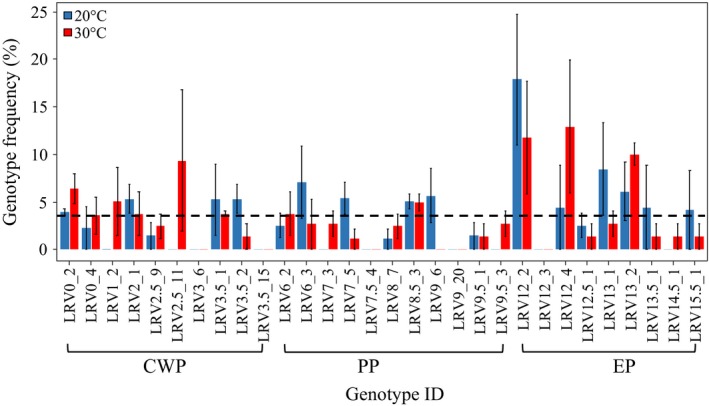
Genotype frequency in the mesocosm competition experiment. Genotype frequencies, with variance among replicate mesocosms, are shown after 4 weeks at 20°C (blue bars) and 30°C (red bars) as compared to an initial equal frequency of genotypes (dotted line). Names on the *x*‐axis are the inoculated genotypes ID, grouped per subpopulation

The initial frequency of genotypes inoculated in the mesocosm experiment did not significantly differ from the frequency of genotypes sampled after 4 weeks of exposure to either a nonstressful temperature regime (20°C) or hyper‐thermal stress (30°C) (Figure 3). In both temperature regimes, the proportion of the three subpopulations shifted in favour of the EP subpopulation, but this shift was not significant [χ^2^_20°C_ (2) 4.93, *p* = .11; (χ^2^_30°C_ (2) 2.58, *p* = .27]. Difference in genotype frequency between the two temperature regimes was also not significant [χ^2^_20°/30°C_ (5) 6.98, *p* = .22]. After 4 weeks of selection, 69% of the inoculated genotypes were recovered in the mesocosm exposed to 20°C and 83% were recovered in the mesocosm exposed to 30°C (Figure 4). These values are compatible with a Poissonian expectation of recovering at least one representative of each genotype in a sample of 32 individuals. The genotypes that maintained a frequency similar to the initial inoculum were 25% of the recovered genotypes at 20°C and 8% of the recovered genotypes at 30°C. However, the number of genotypes occurring with frequency higher than the initial inoculum (>5) was comparable between the experimental temperatures. Nonsignificant difference in genotype frequencies among the temperature regimes was confirmed by a PCA plot showing almost complete overlap of genotype frequency at the two temperature treatments after 4 weeks of selection (Appendix S5). The genotypes with the highest frequency in the mesocosm competition experiment show the highest PC bearings (Appendix S5).

### 3.3 Competitive abilities of the Hb‐rich genotypes after acclimation to hyper‐thermal stress—microcosms

**Figure 5:**
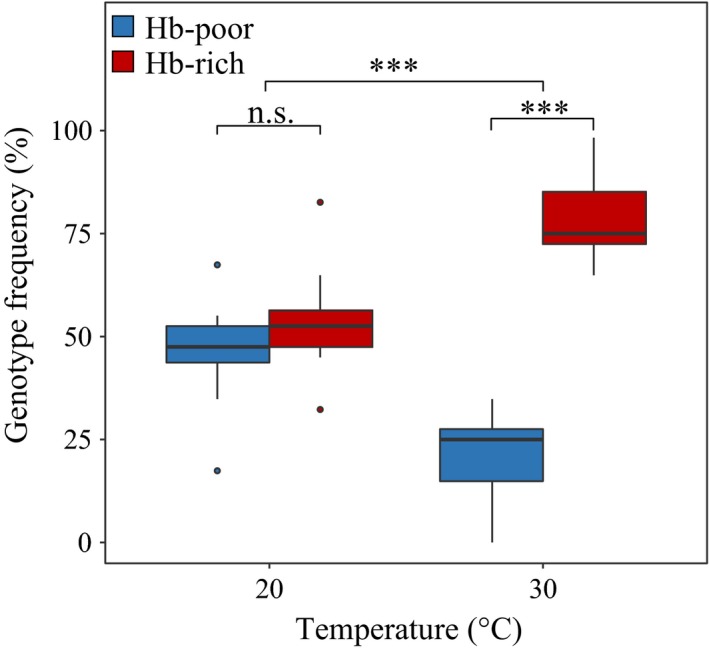
Genotype frequency in the microcosm competition experiment. Genotype frequencies median and quartiles (25th and 75th) based on Hb expression under hyper‐thermal stress (30°C) as compared to a nonstressful temperature regime (20°C). Competition abilities were tested in three random pairs of genotypes diverging in Hb content (Appendix S2). The genotypes were inoculated at equal frequency (50%)

After acclimation for three generations to hyper‐thermal stress, Hb‐rich genotypes always showed a significantly higher competitive performance than Hb‐poor genotypes [χ^2^_30°C_ (1) = 31.94; *p* = 1.6E‐8] (Figure 5), whereas Hb‐poor and Hb‐rich genotypes had comparable competitive abilities at the nonstressful temperature regime [χ^2^_20°C_ (1)^ ^= 0.53; *p* = .47)]. The difference between the two treatments was highly significant [χ^2^_20°/30°C_ (1) = 12.43; *p* = 4.2E‐4] (Figure 5).

### 3.4 Time to immobilization under hyper‐thermal stress

**Figure 6:**
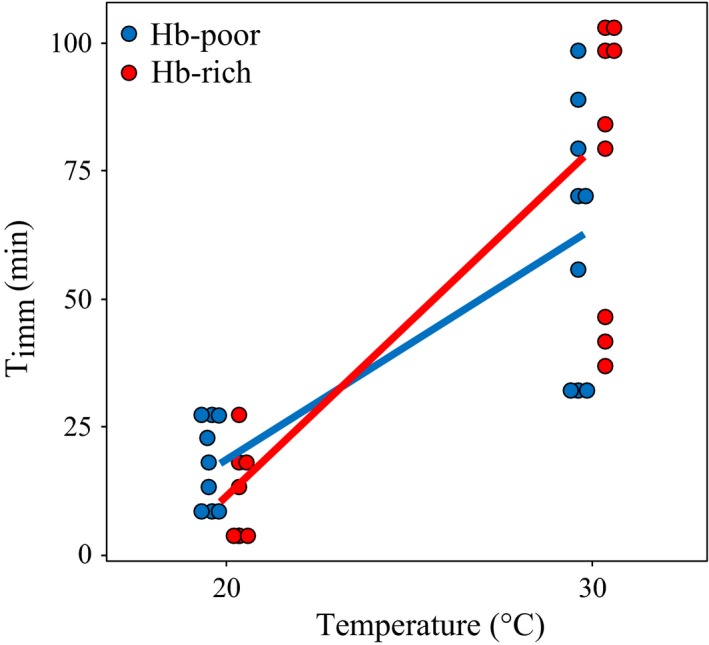
Time until immobilization (*T*_imm_). Time until immobilization (*T*_imm_) as a function of experimental temperature. Animals (triplicates per genotype) acclimated at 20 and 30°C for three generations were exposed to 37°C and time until locomotion was lost was recorded (minutes). The difference in *T*_imm_ between experimental temperatures and between Hb‐rich and Hb‐poor genotypes was tested with a two‐way ANOVA (Appendix S6)

After three generations of acclimation at 30°C, both Hb‐poor and Hb‐rich genotypes show a significant increase in the time to immobilization (*T*_imm_), effectively extending the time locomotion abilities are lost (Figure 6; Appendix S6). However, the time until immobilization does not significantly differ between Hb‐poor and Hb‐rich genotypes within the same temperature regime (Figure 6; Appendix S6).

## 4 DISCUSSION

Our results show that plasticity in Hb expression is a coping mechanism to hyper‐thermal stress in the studied population. Hb‐rich genotypes show superior competitive abilities after acclimation to hyper‐thermal stress, suggesting that long‐term adjustment to higher occurrence of heat waves may require a combination of plasticity and genetic adaptation. Higher occurrence of heat waves may impact local population genetic diversity by favouring Hb‐rich genotypes over evolutionary times.

Numerous studies have suggested that temperature‐dependent performance or thermal tolerance of aquatic invertebrates is shaped by the capacity for oxygen delivery in relation to oxygen demand [reviewed in (Pörtner, 2002; Verberk et al., 2016)]. If correct, oxygen transport provides a mechanistic framework to understand and predict both current and future impacts of rapidly changing climate. Because of the essential role that Hb plays in the oxygen transport system, and the direct link between temperature changes and oxygen solubility, Hb induction has been studied for its link to thermal stress in ectotherms (Pörtner, 2002; Lamkemeyer et al., 2003; Paul, Zeis, et al., 2004; Paul, Lamkemeyer, et al., 2004; Verberk et al., 2016). However, the extent to which the evolutionary response to extreme temperatures is mediated by regulation of this protein is still unknown. This is because the evolution of thermal tolerance, of energy metabolism and of oxygen transport occurs across generations. Moreover, temperature changes co‐vary with other environmental stressors, making it challenging to disentangle the response to temperature increase from the one to other environmental factors.

Here, we disentangle the Hb evolutionary and plastic response in a natural population of *D. magna* through five decades of modest average temperature increase, growing occurrence of heat waves and a transition from high to low primary production levels, affecting oxygen availability. We show that the constitutive protein content does not significantly differ among the subpopulations resurrected along this temporal gradient, whereas all genotypes show a significant plastic response to hyper‐thermal stress. These findings suggest that the modest increase in average temperature occurring over the past five decades did not trigger an evolutionary response in the protein Hb, at least in the population studied here. They also suggest that *Daphnia* has the potential to adjust to the increasing occurrence of heat waves via plasticity.

Plastic mechanisms enable ectotherms to cope with fluctuations in the natural environment and are expected to provide a short‐term fix to cope with fluctuating climatic conditions (Seebacher, Davison, Lowe, & Franklin, 2005), although evolutionary responses are expected to play a stronger role for long‐term persistence (Hoffmann & Sgro, 2011; Jansen et al., 2017; Merila & Hendry, 2014). In line with this expectation, we observe that acclimation to high temperatures only improves competitive abilities of Hb‐rich genotypes. Overall, the competition experiments showed that competitive abilities of Hb‐rich genotypes are superior to the ones of Hb‐poor genotypes under hyper‐thermal stress after acclimation to high temperatures, confirming previous studies that link oxygen regulation to hyper‐thermal stress in laboratory settings (Lamkemeyer et al., 2003; Paul, Zeis, et al., 2004; Paul, Lamkemeyer, et al., 2004; Pörtner, 2002; Verberk et al., 2016). However, these competitive abilities are dampened in absence of acclimation, as previously observed (Seidl, Pirow, & Paul, 2005; Williams, Dick, & Yampolsky, 2012). Overall, these findings supported by a significant interaction between constitutive Hb protein content and population under hyper‐thermal stress (Appendix S3; Pop × Temp), suggest that the interplay between genetic and plastic response may be key to regulate Hb metabolism in *D. magna* over evolutionary times.

It is noteworthy that the natural environment we study here, as many natural environments, is complex as multiple environmental and ecological factors co‐vary with temperature over time. These co‐occurring environmental and ecological variables may indirectly influence oxygen metabolism by altering water chemistry and thus can strongly influence evolutionary responses in natural populations.

Although we cannot exclude that other environmental factors co‐occurring with temperature (average increase and extreme events) may have influenced Hb change over time, our experiments directly link Hb protein content and response to hyper‐thermal stress in nonlimiting oxygen levels.

### 4.1 Evolutionary applications

A thorough understanding of the physiological mechanisms underpinning thermal tolerance of living organisms is essential to predict the impacts of current and future rises in global temperatures, and of extreme temperature events (Verberk et al., 2016). Here, we provide critical insights into the role of Hb as a biomarker in the response to hyper‐thermal stress of a keystone species in freshwater ecosystems. We observe that under persistent hyper‐thermal stress Hb‐rich genotypes have higher competitive abilities than Hb‐poor genotypes, with potential implication for long‐term effects on the genetic composition of local populations. The introduction of Hb‐rich genotypes in more susceptible areas of the globe affected by tropicalization (Jeppesen et al., 2009, 2010) may be a viable strategy to buffer the effect of hyper‐thermal stress on the loss of local genetic diversity (Peretyatko, Teissier, De Backer, & Triest, 2012; Sarnelle, 2007). However, the influence of other environmental stressors on the local environment should be carefully evaluated prior to any translocation.

Current pattern‐related predictions for species survival in the face of future global warming do not take into account ecological and evolutionary processes, limiting our ability to forecast the impacts of climate change on biota (Orsini et al., 2013). Long‐term empirical data have the power to improve the accuracy of forecasting models on which predictions for species persistence are realized (Urban, 2015). As zooplankters have physiological processes highly sensitive to temperature (Lamkemeyer et al., 2003; Mauchline, 1998) and are short‐lived (<1 year), they are better proxies of climate dynamics than climate variables (Hays, Richardson, & Robinson, 2005). Here, we provide empirical evidence of the response to temperature and water chemistry changes over evolutionary times of a central zooplankter in freshwater ecosystems. These data, replicated across multiple environments, are a powerful resource for forecast models to obtain more accurate predictions of species persistence.

## CONFLICT OF INTEREST

The authors declare no conflict of interest.

## DATA ARCHIVING

Data for this study are available at the Dryad Digital Repository: https://doi.org/10.5061/dryad.5k6t6.
