# Supplementary material for: Haemoglobin‐mediated response to hyper‐thermal stress in the keystone species Daphnia magna
Source: Evol Appl. 2017 Nov 2;11(1):112–20. doi: 10.1111/eva.12561 (PMC5748520; doi:10.1111/eva.12561)
Supplement: Supplementary file 1 [file EVA-11-112-s001.docx]

**Haemoglobin-mediated response to hyper-thermal stress in the keystone species *Daphnia magna***

Running head: Hyper-thermal stress response in *Daphnia*

**Appendices**

**Appendix 1. Microsatellite multiplexes.**

The NCBI Accession Number (AN), the multiplex information, the PCR primer sequences, the PCR size range, the repeat motif, the dye used to label the forward primer, the annealing temperature (Tm) and the multiplex (M) are shown.

| Locus | AN | Size range (bp) | Primers (5’-3’) | Dye label | Repeat motif | Tm | M |
| --- | --- | --- | --- | --- | --- | --- | --- |
| B008 | HQ234154 | 150-170 | F: TGGGATCACAACGTTACACAA  R: GCTGCTCGAGTCCTGAAATC | VIC | (TC)^9^ | 56.0 | 1 |
| B030 | HQ234160 | 154-172 | F: CCAGCACACAAAGACGAA  R: ACCATTTCTCTCCCCCAACT | PET | (GA)^11^ | 56.0 | 1 |
| B045 | HQ234168 | 118-126 | F: GCTCATCATCCCTCTGCTTC  R: ATAGTTTCAGCAACGCGTCA | NED | (TG)^8^ | 56.0 | 1 |
| B050 | HQ234170 | 234-248 | F: TTTCAAAAATCGCTCCCATC  R: TATGGCGTGGAATGTTTCAG | 6FAM | (GAA)^6^ | 56.0 | 1 |
| B064 | HQ234172 | 135-151 | F: CTCCTTAGCAACCGAATCCA  R: CAAACGCGTTCGATTAAAGA | 6FAM | (TC)^8^ | 56.0 | 1 |
| B074 | HQ234174 | 196-204 | F: TCTTTCAGCGCACAATGAAT  R: TGTGTTCCTTGTCAACTGTCG | NED | (GT)^9^ | 56.0 | 1 |
| B096 | HQ234181 | 234-240 | F: GGATCTGGCAGGAAGTGGTA  R: TTGAACCACGTCGAGGATTT | VIC | (AC)^15^ | 56.0 | 1 |
| B107 | HQ234184 | 250-274 | F: GGGGTGAAGCATCAAAGAAA  R: TGTGACCAGGATAAGAGAAGAGG | PET | (CT)^8^ | 56.0 | 1 |
| B087 | HQ234178 | 174-200 | F: CGAATTCGTTTGTCTGATAGGG | 6FAM | (CA)^13^ | 54.0 | 5 |
|  |  |  | R: CCTAACAGTCGCGTCATTCA |  |  |  |  |
| A002 | HQ234126 | 250-282 | F: GTTCCACAGATAGACATTTGCT | 6FAM | (AG)^8^ | 54.0 | 5 |
|  |  |  | R: GTTGAAATGCAAATGAGTCG |  |  |  |  |
| B052 | HQ234171 | 277-305 | F: AAGCTTGGGATCGTCTGCT | PET | (CA)^12^ | 54.0 | 5 |
|  |  |  | R: CGAGATTTGGTGTGTGATGG |  |  |  |  |
| B180 | HQ234207 | 301-311 | F: CAGCATCGCTCTGTAACTCG | VIC | (GA)^8^ | 54.0 | 5 |
|  |  |  | R: GGATTTTCATGACCGGCTTA |  |  |  |  |
| B033 | HQ234163 | 96-114 | F: AGGCATTCCTCAATTTCCAA | NED | (TG)^9^ | 54.0 | 5 |
|  |  |  | R: GAAGACGGCGTGGTTAGTTT |  |  |  |  |

**Appendix 2**. **Haemoglobin content in crude extracts at 30°C.**

Distribution of haemoglobin content measured at 30°C in the 30 genotypes resurrected from the sedimentary archive of Lake Ring. Genotypes in black are used in the microcosm competition experiment and in the T_imm_ assays. For a complete list of Hb content at both experimental temperatures see Appendix 4.

**
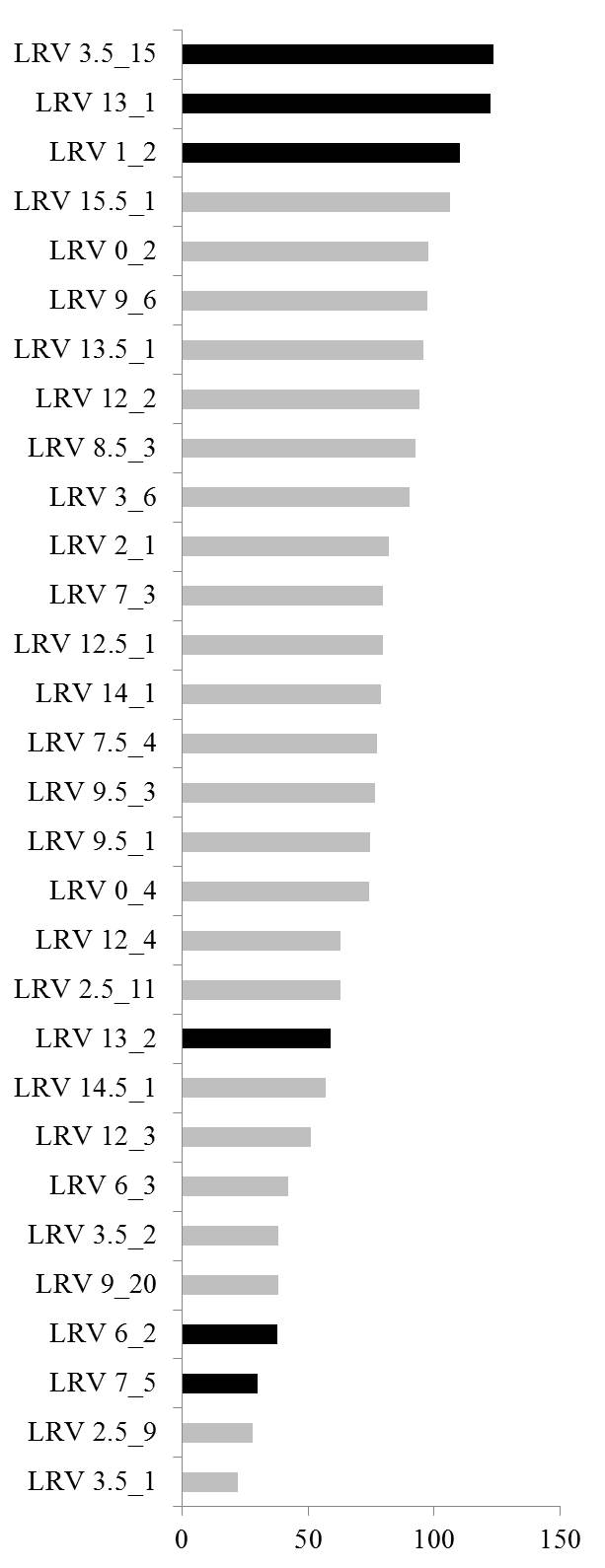
**

**Appendix 3. Evolutionary and plastic response to hyper-thermal stress**

Analysis of variance testing whether changes in haemoglobin content (response variable) of the resurrected (sub)populations can be explained by evolution (constitutive differences in Hb expression among (sub)populations - Pop), plasticity (differences among temperatures - Temp) or their interaction term. Genotype was included as random variable nested within population. Significant p-values (P<0.05) are shown in bold.

|  | Df | SS | *p-value* |
| --- | --- | --- | --- |
| (sub)population (Pop) | 2 | 212.9 | 0.69 |
| Temperature (Temp) | 1 | 17131.6 | **<0.0001** |
| Pop x Temp | 2 | 2216.2 | **0.05** |

**Appendix 4**. **Haemoglobin content under experimental conditions**

Haemoglobin (Hb) content measured on the crude extract of 30 genotypes resurrected from Lake Ring under non-stressful temperature (20°C) and hyper-thermal stress (30°C). Values of Hb are shown for three replicates per condition in µmol/L. Average across replicates within temperature (mean) and log2 fold change between the mean values are also shown. An asterisk (*) indicates genotypes with the highest frequency in the mesocosm competition experiment (Fig. 3). Genotypes ID are as in (Orsini et al. 2016).

|  | 20°C |  |  |  | 30°C |  |  |  |  |
| --- | --- | --- | --- | --- | --- | --- | --- | --- | --- |
| genotypeID | Hb (µmol/L) | Hb (µmol/L) | Hb (µmol/L) | mean | Hb (µmol/L) | Hb (µmol/L) | Hb (µmol/L) | mean | log2-fold change |
| LRV 0_2* | 79.98 | 70.64 | 62.42 | 71.01 | 87.58 | 113.96 | 91.27 | 97.60 | 0.46 |
| LRV 0_4 | 45.10 | 62.42 | 64.37 | 57.30 | 88.30 | 41.89 | 92.71 | 74.30 | 0.37 |
| LRV 1_2* | 26.70 | 35.52 | 44.87 | 35.70 | 146.40 | 65.61 | 118.28 | 110.09 | 1.62 |
| LRV 2_1 | 70.40 | 59.14 | 55.65 | 61.73 | 109.20 | 61.40 | 75.77 | 82.12 | 0.41 |
| LRV 2.5_9 | 26.69 | 20.12 | 19.40 | 22.07 | 25.15 | 30.70 | 28.54 | 28.13 | 0.35 |
| LRV 2.5_11* | 52.36 | 44.87 | 34.19 | 43.81 | 47.64 | 63.35 | 77.00 | 62.66 | 0.52 |
| LRV 3_6 | 46.40 | 54.00 | 46.10 | 48.83 | 109.50 | 74.13 | 87.37 | 90.33 | 0.89 |
| LRV 3.5_1 | 25.30 | 2.71 | 11.75 | 13.25 | 26.88 | 12.88 | 26.88 | 22.21 | 0.75 |
| LRV 3.5_2 | 48.25 | 39.84 | 42.30 | 43.46 | 45.69 | 29.16 | 39.73 | 38.19 | -0.19 |
| LRV3.5_15 | 99.18 | 65.71 | 76.90 | 80.60 | 122.48 | 135.73 | 112.63 | 123.61 | 0.62 |
| LRV 6_2 | 74.02 | 71.36 | 68.48 | 71.29 | 39.12 | 43.12 | 30.80 | 37.68 | -0.92 |
| LRV 6_3 | 64.40 | 47.33 | 48.25 | 53.33 | 59.30 | 29.16 | 38.40 | 42.29 | -0.33 |
| LRV 7_3 | 61.90 | 41.48 | 63.86 | 55.75 | 93.80 | 53.18 | 92.61 | 79.86 | 0.52 |
| LRV 7_5 | 68.89 | 50.62 | 48.87 | 56.13 | 44.25 | 22.48 | 23.61 | 30.12 | -0.90 |
| LRV 7.5_4 | 61.50 | 61.19 | 79.26 | 67.32 | 71.87 | 77.21 | 83.37 | 77.48 | 0.20 |
| LRV 8.5_3 | 55.95 | 46.30 | 75.05 | 59.10 | 64.17 | 102.36 | 110.88 | 92.47 | 0.65 |
| LRV 9_6 | 56.90 | 39.53 | 29.36 | 41.93 | 83.80 | 100.92 | 107.29 | 97.34 | 1.22 |
| LRV 9_20 | 57.91 | 48.67 | 42.30 | 49.62 | 50.92 | 30.08 | 33.26 | 38.09 | -0.38 |
| LRV 9.5_1 | 42.51 | 44.56 | 59.14 | 48.73 | 47.74 | 86.65 | 90.14 | 74.85 | 0.62 |
| LRV 9.5_3 | 87.78 | 91.48 | 74.13 | 84.46 | 55.44 | 87.47 | 87.17 | 76.69 | -0.14 |
| LRV 12_2* | 67.20 | 57.91 | 33.26 | 52.79 | 101.10 | 88.19 | 92.92 | 94.07 | 0.83 |
| LRV 12_3 | 56.88 | 35.01 | 28.85 | 40.25 | 97.43 | 24.85 | 31.01 | 51.10 | 0.34 |
| LRV 12_4* | 33.47 | 50.62 | 58.01 | 47.36 | 52.46 | 77.93 | 57.91 | 62.77 | 0.41 |
| LRV12.5_1 | 42.51 | 71.97 | 64.78 | 59.75 | 57.39 | 93.84 | 88.19 | 79.81 | 0.42 |
| LRV 13_1 | 59.20 | 51.95 | 35.32 | 48.82 | 115.20 | 112.94 | 138.91 | 122.35 | 1.33 |
| LRV 13_2* | 16.94 | 26.88 | 25.07 | 22.96 | 63.47 | 54.89 | 58.95 | 59.10 | 1.36 |
| LRV 13.5_1 | 53.70 | 55.44 | 76.39 | 61.84 | 73.31 | 110.37 | 104.11 | 95.93 | 0.63 |
| LRV 14_1 | 73.30 | 51.95 | 68.58 | 64.61 | 64.80 | 80.90 | 90.66 | 78.79 | 0.29 |
| LRV 14.5_1 | 82.55 | 54.21 | 46.10 | 60.95 | 57.60 | 49.18 | 63.86 | 56.88 | -0.10 |
| LRV 15.5_1 | 60.80 | 69.51 | 48.15 | 59.49 | 95.50 | 115.81 | 107.39 | 106.23 | 0.84 |

**Appendix 5. PCA plot**

PCA plot of genotype frequency changes after exposure to 20°C (blue) and 30°C (red) for four weeks as compared to the starting inoculum (start), in which an equal number of clones per genotype were inoculated.

**
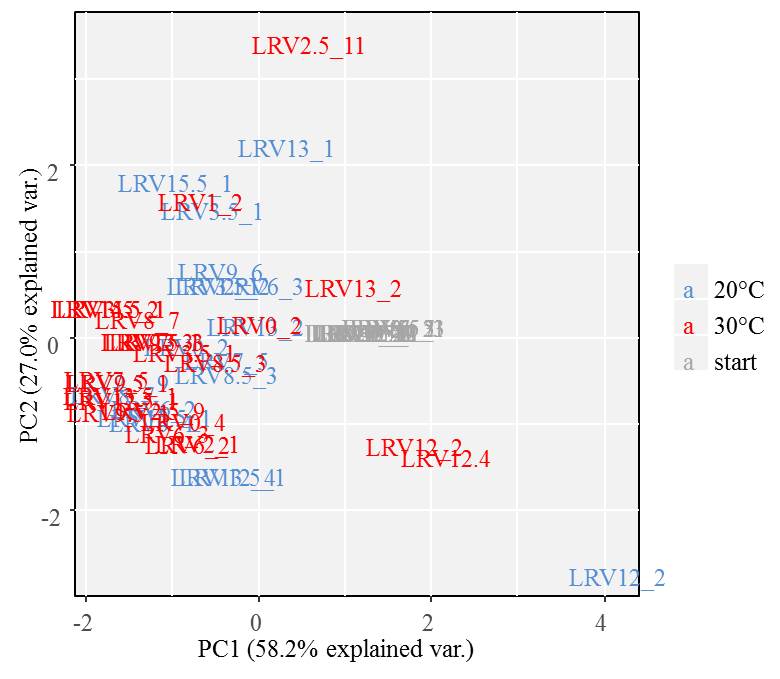
**

**Appendix 6. Time to immobilization.**

Analysis of variance testing whether the time to immobilization (temperature tolerance as knockout time) is explained by constitutive differences in haemoglobin (Hb) expression (Hb-rich vs Hb-poor genotypes expression), the treatment (experimental temperature) and their interaction term. Significant p-values (P<0.05) are shown in bold.

|  | Df | SS | p-value |
| --- | --- | --- | --- |
| Hb (poor/rich) | 1 | 136.1 | 0.59 |
| Treatment (Temp) | 1 | 30625 | **2.11E-9** |
| Hb x Temp | 1 | 1225 | 0.10 |
